# Darstellung der eosinophilen Fasziitis in Ultraschall und MRT (Magnetresonanztomographie): ein Fallbericht

**DOI:** 10.1007/s00393-022-01207-3

**Published:** 2022-05-16

**Authors:** Nicolas Gerritzen, Jana Ziob, Peter Brossart, Valentin S. Schäfer

**Affiliations:** 1 Medizinische Klinik III, Onkologie, Hämatologie, Rheumatologie und klinische Immunologie, Universitätsklinikum Bonn, Venusberg Campus 1, 53127 Bonn, Deutschland; 2 Klinik und Poliklinik für Dermatologie und Allergologie, Universitätsklinikum Bonn, Bonn, Deutschland

**Keywords:** Sonographie, Shulman-Syndrom, Groove-Sign, Verdickung der Faszien, Eosinophilie, Sonography, Shulman syndrome, Groove sign, Thickening of the fascia, Eosinophilia

## Abstract

Die eosinophile Fasziitis (EF, auch Shulman-Syndrom) ist eine seltene Erkrankung des Bindegewebes mit entzündlicher Verdickung der Faszien sowie Schwellung und Verhärtung der Haut. Betroffen sind v. a. die distalen Extremitäten. Typische klinische Befunde stellen eine lokalisierte Schwellung und Verhärtung der Haut sowie das Groove-Sign (deutsch: Rillenzeichen/negatives Venenzeichen/Matratzenphänomen) dar. Der Goldstandard für die Diagnosesicherung ist bisher der bioptische Nachweis entzündlich verdickter Faszien. In der Literatur wird alternativ die Diagnosesicherung durch MRT-Bildgebung diskutiert. Wir berichten über einen Fall von asymmetrischer EF bei einem 54 Jahre alten, männlichen Deutschen. Die Vorstellung erfolgte mit schmerzhafter Verhärtung im Bereich des rechten Unterarms sowie charakteristischem Groove-Sign und einer Bewegungseinschränkung der rechten Hand. Im Blutbild zeigte sich eine Eosinophilie mit 0,57 G/l bzw. 9,6 % (norm. 0,05–0,5 G/l und 0,5–5,5 %), ANAs und ENAs waren negativ. Die Diagnosesicherung erfolgte histologisch, zusätzlich konnten wir verdickte Faszien sowohl im MRT als auch in der Sonographie nachweisen. Im Verlauf manifestierte sich die EF auch am linken Malleolus lateralis. Die Therapie erfolgte mit Prednisolon und Methotrexat.

## Abstract (en)

Eosinophilic fasciitis (EF, also known as Shulman syndrome) is an uncommon connective tissue disease characterized by inflammatory thickening of the fasciae as well as swelling and hardening of the skin. It mostly affects the lower extremities. Swollen and indurated skin, together with the groove sign, are typical clinical signs. So far, biopsy evidence of inflammation and thickening of the fascia has been the gold standard for diagnosis. Magnetic resonance imaging (MRI) is mentioned in the literature as an alternative method for confirming the diagnosis. We present a case of asymmetric EF in a 54-year-old German male. He came with painful induration of the right forearm, with a characteristic groove sign and limitation of motion of the right hand. The blood count revealed eosinophilia with 0.57 G/l or 9.6% (normal: 0.05–0.5 G/l and 0.5–5.5%), ANA and ENA were negative. The diagnosis was confirmed histologically and we were able to detect a thickened fascia in MRI and ultrasound imaging. The EF also appeared in the left lateral malleolus during the course of the illness. Treatment was carried out with prednisolone and methotrexate.

Die eosinophile Fasziitis (früher: Shulman-Syndrom) ist eine seltene Bindegewebserkrankung, eine Sonderform der zirkumskripten Sklerodermie, die sich v. a. an den distalen Extremitäten in Form einer entzündlichen Verdickung der Faszien und Schwellung sowie Verhärtung der darüber liegenden Hautstrukturen manifestiert. Die Diagnosestellung erfolgt bisher in der Regel durch eine tiefe Hautbiopsie bis auf den Muskel mit histologischem Nachweis einer entzündlichen Verdickung der Faszien. Der Nachweis einer Eosinophilie im Gewebe oder im peripheren Blutbild ist oft nur in den frühen Stadien der Erkrankung möglich. Die eosinophile Fasziitis spricht in der Regel gut auf Kortikosteroide an. Als Kortikosteroid-sparende Langzeittherapie wurde in den meisten berichteten Fällen Methotrexat eingesetzt.

## Fallbeschreibung

### Anamnese

Ein 54-jähriger männlicher Patient stellt sich mit seit 9 Monaten bestehenden deutlichen Bewegungseinschränkungen der rechten Hand und einer schmerzhaften Verhärtung im Bereich des rechten anterioren Unterarmes, distal betont, in der Rheumatologie am Universitätsklinikum Bonn vor. Die Vorstellung erfolgt konsiliarisch über die Dermatologie bei Verdacht auf Shulman-Syndrom.

Der Patient ist gelernter Möbelmonteur, aktuell im Versandhandel tätig und gibt an, häufig Bewegungen mit monotoner Belastung der Unterarmmuskulatur auszuführen.

Bereits 2 Jahre zuvor trat eine ähnliche, jedoch oberflächliche Verhärtung im Bereich der rechten Skapula auf. Diese zeigte initial das Bild einer bräunlich indurierten Plaque mit zentraler Abblassung und Atrophie. Die behandelnden Ärzte äußerten den Verdacht einer limitierten Form der zirkumskripten Sklerodermie (Morphea, Plaquetyp). Histologisch zeigten sich eine verstärkte Fibrose und ein perivaskuläres lymphozytäres Infiltrat in der Dermis, und damit ein mit der klinischen Verdachtsdiagnose vereinbarer Befund. Zur Therapie wurde intravenös Penicillin V verabreicht. (Die Wirksamkeit von Penicillin V bei zirkumskripter Sklerodermie ist umstritten. Der Einsatz von Penicillin V wird in den aktuell gültigen Leitlinien nicht mehr empfohlen [1]).

Aufgrund der Vordiagnose einer zirkumskripten Sklerodermie hat der behandelnde Hausarzt den Verdacht auf eine erneute Manifestation der Sklerodermie geäußert und den Patienten an die Dermatologie des Universitätsklinikums Bonn überwiesen. Im Rahmen eines kurz zuvor durchgeführten orthopädischen Konsils wurde der Verdacht auf ein Karpaltunnelsyndrom geäußert, ausgelöst durch ein mutmaßliches Rezidiv der zirkumskripten Sklerodermie. Zur Mitbeurteilung und Koordination der Behandlung wurde der Patient in der Rheumatologie vorgestellt.

### Klinische Untersuchung

**Figure 1:**
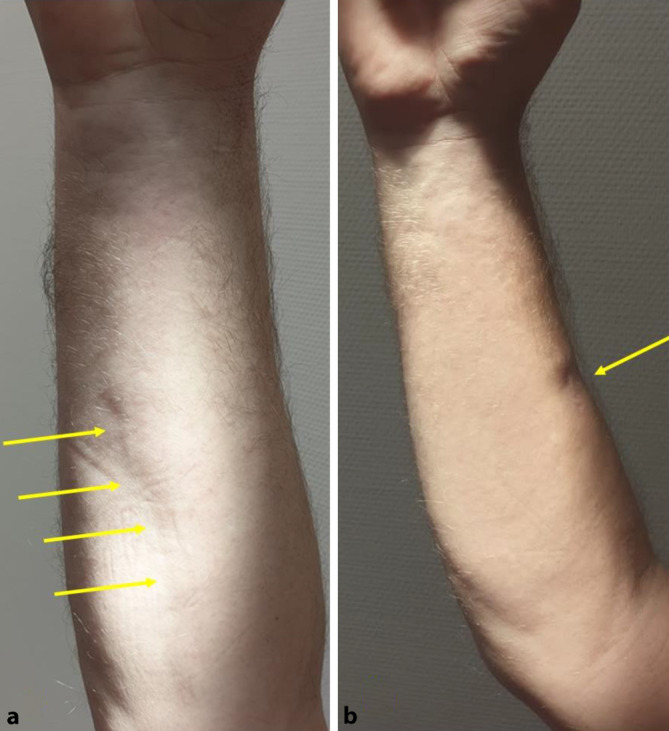

In der klinischen Untersuchung zeigte sich eine tastbare Verhärtung subkutan im Bereich des anterioren Unterarms rechts mit Groove-Sign (Abb. 1), einer geradlinigen Einsenkung entlang des Verlaufs der oberflächlichen Unterarmvenen, verstärkt bei Elevation (Abb. 1a). Im Bereich der rechten Skapula zeigte sich eine handtellergroße, blass erythematöse teils bräunliche Makula mit zentraler Atrophie.

Die Beweglichkeit im rechten Handgelenk war eingeschränkt (E/F 30°–0°–40°) bei normaler Beweglichkeit der Gegenseite. Bei Extension trat ein schmerzhaftes Spannungsgefühl auf, insbesondere im Bereich der daumenseitigen Flexorenloge. Eine Atrophie der Unterarmmuskulatur bestand nicht. Der Patient berichtete über Arthralgien im rechten Ellenbogen. Der Patient gab keine B-Symptomatik an. Eine Raynaud-Symptomatik bestand nicht.

### Labor

Im Labor zeigte sich eine Eosinophilie (0,57 G/l und 9,6 %, norm. 0,05–0,5 G/l und 0,5–5,5 %) und ein leicht erhöhtes CRP (8,96 mg/l, norm. < 3 mg/l). Der ANA-Titer war mit 1:80 (norm. < 1:80) grenzwertig, die Scl-70-Antikörper waren im Immunoblot leichtgradig positiv, im ELISA-Verfahren allerdings negativ. Im restlichen Autoimmunlabor zeigten sich keine Auffälligkeiten.

### Bildgebung

**Figure 2:**
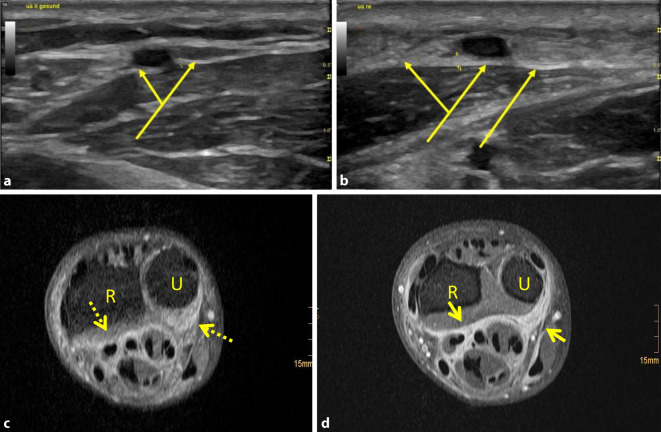

Ein im Vorfeld ambulant durchgeführtes MRT (Abb. 2c, d) des rechten Unterarms zeigte eine chronisch entzündliche Fasziitis – mit Betonung der Flexorenloge und diffuser Verdickung der Faszie des anterioren Kompartiments sowie eine durch Weichteilvermehrung ausgelöste Schwellung und Distanzierung der einzelnen Muskelgruppen.

In der hochauflösenden Sonographie mit einem 18 MHz Hockey-Stick des rechten Unterarms (Abb. 2b) zeigte sich ein typischer Befund verdickter Faszien im Bereich der beschriebenen Verhärtung am rechten Unterarm. Zum Vergleich zeigt Abb. 2a den unauffälligen linken Unterarm.

Es gab keinen Anhalt für eine mögliche Organbeteiligung. Eine Röntgenaufnahme des Thorax war unauffällig. Eine im Verlauf durchgeführte Sonographie des Abdomens zeigte bis auf eine leichte Hepatomegalie (17,4 cm Durchmesser in der Medioclavicularlinie) und Steatosis hepatis einen unauffälligen Befund.

### Histologie

Es wurde eine tiefe Hautbiopsie bis auf die Muskelfaszie durchgeführt. Hier zeigte sich eine korbgeflechtartige Orthokeratose über schmalem Epithel, das darunterliegende obere Korium war unauffällig. Im tieferen Korium, der Subkutis sowie im Bereich der angeschnittenen Faszienanteile zeigte sich ein herdförmiges, überwiegend perivaskulär akzentuiertes, dichtes, gemischtzelliges entzündliches Infiltrat mit Lymphozyten und Histiozyten sowie Clustern von Mastzellen und kräftigen Muzinablagerungen.

### Diagnose

In Zusammenschau des klinischen Bildes mit typischem Groove-Sign, des histologischen Befundes, der Darstellung verdickter Faszien in der Sonographie und in der MRT-Bildgebung sowie der peripheren Eosinophilie im Blutbild wurde die Diagnose einer atypischen (weil asymmetrisch) eosinophilen Fasziitis gestellt. Eine Kollagenose oder systemische Sklerose wurde aufgrund des Befallsmusters mit auf die Faszien konzentrierter Entzündung, fehlendem Raynaud-Phänomen, unauffälliger Antikörperdiagnostik sowie fehlenden klinischen Zeichen ausgeschlossen.

In Abgrenzung zur eosinophilen Fasziitis ist die systemische Sklerose nicht auf Haut und Faszien beschränkt, sondern präsentiert sich typischerweise mit positivem Raynaud-Phänomen (durch Gefäßbeteiligung), Sklerodaktylie mit Fingerkruppennekrosen (die Finger sind von der eosinophilen Fasziitis nicht betroffen), Teleangiektasie, Organbefall (Lunge, Niere, Gastrointestinaltrakt) und auffälliger Antikörperdiagnostik (ANA, Anti-ScL-70-AK [Anti-Topoisomerase-I-AK]), Anti-Zentromer-AK, Anti-RNA-Polymerase III-AK [2].

Eine weitere Differenzialdiagnose stellt die „disabling (pansclerotic) morphea“, eine andere Sonderform der zirkumskripten Sklerodermie, dar. Hier kommt es typischerweise zu asymmetrischem, meist streckseitigem Befall von Dermis und Subkutis. Sehnen und Knochen können auch betroffen sein. Der Erkrankungsbeginn liegt meist jedoch vor dem 14. Lebensjahr, einzelne Erkrankungsfälle von Erwachsenen sind beschrieben [3].

### Therapie und Verlauf

Die Therapie erfolgte initial mit einer Kombination aus 15 mg Methotrexat (MTX) subkutan pro Woche und 80 mg Prednisolon pro Tag, überbrückend bis zum vollständigen MTX-Wirkeintritt. Die Prednisolon-Dosis wurde schrittweise, über ca. 5 Monate, bis zum vollständigen Absetzen reduziert.

Die erste Kontrollvorstellung erfolgte ca. 3 Monate nach Therapiebeginn. Der Befund am rechten Unterarm zeigte sich stabil. Der Patient berichtete, die Haut am rechten Unterarm sei geringfügig weicher, die Beweglichkeit des rechten Handgelenkes und der Hand habe sich nicht gebessert. Die Prednisolon-Restdosis betrug zu diesem Zeitpunkt 10 mg/Tag.

Bei der nächsten Kontrollvorstellung 6 Monate nach Therapiebeginn zeigte sich eine Progredienz der eosinophilen Fasziitis. Es fiel eine neu aufgetretene Verhärtung über dem Malleolus lateralis links mit leicht eingeschränkter Beweglichkeit des Fußes auf. Der Befund am rechten Unterarm war weiterhin stabil, bei einer leicht gebesserten Induration der Haut und nicht mehr sichtbarem Groove-Sign (Abb. 1b). Von einer Biopsie der Verhärtung im Bereich des Malleolus lateralis wurde aufgrund eingeschränkter Möglichkeiten zum primären Wundverschluss und zu erwartenden Wundheilungsverzögerungen der indurierten Haut abgesehen. Als Reaktion auf die Progredienz der Erkrankung wurde die MTX-Dosis auf 20 mg/Woche subkutan eskaliert. Zwei Monate später (8 Monate nach initialem Therapiebeginn) wurde die MTX-Dosis bei leichter Progredienz der Verhärtung am linken Malleolus lateralis auf 25 mg MTX 1‑mal/Woche erhöht. Hierunter kam es zu einer Stabilisierung der Erkrankung; 15 Monate nach Diagnosestellung und initialem Therapiebeginn ist die eosinophile Fasziitis weiterhin stabil. Die Verhärtung im Bereich des rechten Unterarms ist spürbar weicher geworden.

## Diskussion

Die eosinophile Fasziitis ist eine seltene Bindegewebserkrankung, erstbeschrieben durch Shulman im Jahr 1974. Es gibt ca. 100 beschriebene Fälle weltweit, v. a. aus Japan. Eine Studie aus dem Jahr 2018 schätzte die Prävalenz auf 14 (95 %-KI 10–21) pro 1 Mio. [4]. Viele Experten ordnen die eosinophile Fasziitis als Sonderform der zirkumskripten Sklerodermie ein, die am ehesten ins Spektrum einer generalisierten zirkumskripten Sklerodermie eingeordnet werden kann [1]. In der Regel zeigt sich ein symmetrisches Erkrankungsmuster mit Schwellung und Induration der distalen Extremitäten durch tiefer gelegene Fibrosierungsprozesse im Bereich der Faszie und Subkutansepten [1]. Die Erkrankung kann sich auch auf die proximalen Extremitäten ausbreiten. Gesicht und Finger sind nicht betroffen.

Das Groove-Sign (deutsch: Rillenzeichen s. oben) stellt einen typischen klinischen Befund einer eosinophilen Fasziitis dar. In den verhärteten Bereichen bleiben lediglich die Blutgefäße weich [5], dadurch kommt es zu einer geradlinigen Einsenkung entlang des Verlaufes der oberflächlichen Venen, welche durch das Anheben der betroffenen Extremität verstärkt wird [6].

Als Goldstandard zur Diagnosesicherung wird derzeit eine tiefe Biopsie der Haut unter Mitnahme der Muskelfaszie betrachtet [5, 7]. Histologisch zeigt sich eine Hypertrophie der Faszie mit Infiltration von Lymphozyten und Plasmazellen, wobei sich die fibrotischen Veränderungen bis in die Dermis ausbreiten können.

In der Literatur wird als Alternative zur bioptischen Diagnosesicherung die Darstellung verdickter Faszien im MRT diskutiert [7]. Uns gelang zusätzlich der sonographische Nachweis verdickter Faszien. Die Sonographie hat sich bereits auf vielen Feldern als eine schnellere, kostengünstigere, noninvasive und ubiquitär verfügbare Alternative zur MRT-Diagnostik oder zur Histologie bewiesen und könnte in Zukunft die Diagnosestellung einer eosinophilen Fasziitis vereinfachen.

Eong et al. beschrieben eine fehlende Kompressibilität der dargestellten verdickten Faszien und zeigten eine Steigerung der Kompressibilität im Rahmen der Therapie mit Prednisolon [8]. Zusätzlich beschrieben Eong et al. eine linear erhöhte Echogenität an beiden Grenzen der verdickten Faszienschicht und bezeichneten diese als echoreiches „Tram-track“-Phänomen [8].

Die aktuelle Datenlage zur Darstellung der eosinophilen Fasziitis in der Sonographie und im MRT ist jedoch noch nicht ausreichend, um zur definitiven Diagnosesicherung auf eine Biopsie mit anschließender histologischer Untersuchung verzichten zu können. Um in Zukunft invasive und aufwendige Diagnostik vermeiden zu können, ist weitere Forschung notwendig.

Eine Arbeitsgruppe der Japanese Dermatological Association hat im Dezember 2017 die bisher einzige Richtlinie mit Diagnosekriterien für die eosinophile Fasziitis publiziert. Nach dieser kann eine Diagnose gestellt werden, wenn das Hauptkriterium und mindestens 1 Nebenkriterium (Tab. 1) erfüllt sind. Für die Diagnosestellung werden als Hauptkriterium symmetrische, plattenförmige sklerotische Läsionen an allen 4 Extremitäten vorausgesetzt [9]. Wir kritisieren, dass Fälle mit asymmetrischem oder noch nicht vollständig ausgeprägtem Krankheitsbild in der Richtlinie nicht berücksichtigt werden. Des Weiteren kritisieren wir, dass Nebenkriterium 1 für die bioptische Diagnosesicherung den Nachweis einer Gewebeeosinophilie fordert, obwohl Gewebe- oder periphere Eosinophilie häufig nur in den früheren Stadien der Erkrankung nachweisbar sind [5].

**Table 1:** 

Hauptkriterium	Symmetrische, indurierte, sklerotische Läsionen an den 4 Gliedmaßen
	Fehlendes Raynaud-Phänomen
	Ausschluss systemische Sklerose
Nebenkriterium 1	Fibrose des subkutanen Bindegewebes mit Verdickung der Faszie und zelluläre Infiltration von Eosinophilen und Monozyten in der Histologie einer Hautbiopsie mit Einbeziehung der Faszie
Nebenkriterium 2	Die Verdickung der Faszie ist in einem bildgebenden Verfahren, wie z. B. der Magnetresonanztomographie (MRT), darstellbar

Einige Autoren empfehlen eine alleinige Therapie mit Prednisolon, da hierfür bereits langjährige Erfahrungswerte vorliegen [5, 9, 10]. Wir entschieden uns für eine Cortison-sparende Langzeittherapie mit Methotrexat (MTX), um eine Prednisolon-Dauertherapie zu vermeiden. Neue Erkenntnisse legen zudem nahe, dass unter einer initialen Kombinationstherapie aus oralen Glukokortikoiden und MTX die Remissionsrate höher ist als unter einer alleinigen Glukokortikoid-Therapie [11]. Als Alternative zu MTX wurde in einzelnen Fällen der erfolgreiche Einsatz von Hydroxychloroquin, Sulfasalazin oder Cyclosporin berichtet [10].

## Fazit für die Praxis

- Die eosinophile Fasziitis ist eine nur selten beschriebene Erkrankung, deren Diagnose und Behandlung eine Herausforderung darstellt.
- Das „Groove-Sign“ stellt einen typischen Befund der eosinophilen Fasziitis dar und kann bereits in der klinischen Untersuchung den entscheidenden Hinweis für die Diagnosestellung liefern.
- Bildgebende Verfahren (wie MRT und Sonographie) können eine schnelle und noninvasive Alternative zur histologischen Diagnosesicherung darstellen.
